# Alloying Elements Effects on Electrical Conductivity and Mechanical Properties of Newly Fabricated Al Based Alloys Produced by Conventional Casting Process

**DOI:** 10.3390/ma14143971

**Published:** 2021-07-16

**Authors:** Hany S. Abdo, Asiful H. Seikh, Jabair Ali Mohammed, Mahmoud S. Soliman

**Affiliations:** Mechanical Engineering Department, King Saud University, P.O. Box 800, Al-Riyadh 11421, Saudi Arabia; habdo@ksu.edu.sa (H.S.A.); jmohammed@ksu.edu.sa (J.A.M.); solimanm@ksu.edu.sa (M.S.S.)

**Keywords:** aluminum alloys, casting, heat treatment, conductivity, mechanical strength

## Abstract

The present investigation deals with a comprehensive study on the production of aluminum based alloys with the incorporation of different alloying elements and their effect on its electrical conductivity and mechanical properties. Casting of pure aluminum with different concentration and combinations of alloying additives such as cupper (Cu), magnesium (Mg) and silver (Ag) were carried out using a graphite crucible. The as-cast microstructure was modified by hot rolling followed by different heat-treated conditions viz., annealing, normalizing, quenching, and age hardening. The mechanical properties and electrical conductivity of the produced heat-treated alloys sheets under various processing conditions were carried out using tensile testing, hardness, and electrical resistivity measurements. It was found that by increasing the alloying elements content, yield strength results increased significantly by more than 250% and 500% for the as rolled and 8 h aged Al-Cu-Mg alloy, respectively. On the other hand, the electrical conductivity reduces slightly with −14.6% and −16.57% for the as rolled and 8 h aged of the same Al-Cu-Mg alloy, respectively.

## 1. Introduction

Aluminum and its alloys have very wide applications in a variety of fields such as aircrafts, aerospace, vehicles, electricity, building, packaging, electronic, and kitchen utensils etc.), mostly due to its light weight, corrosion resistance, and good electrical and mechanical properties. The high strength to mass (strength/weight) ratio of aluminum alloys is superior to that of almost all other engineering materials. Aluminum is classified as a light metal and its strength can be improved by alloying, mechanical, and heat treatment, thereby improving its mechanical properties [1,2]. Presently, researchers and scientists worldwide have focused their attention on improving both the mechanical and electrical properties of aluminum for its use in a wide range of applications.

From the last two decades, due to the increase in demand from the electricity transmission lines, copper is being replaced by Al because of its light weight and relatively low cost [3]. In addition, among the engineering conductor materials, aluminum has a very good electrical conductivity due to lower specific gravity (almost in the second rank after copper). The replacing of copper by aluminum for electricity transmission by overhead ACSR (aluminum conductor steel-reinforced) conductors, power cables, etc. has increased all over the world. Moreover, substitution of copper by aluminum also takes place in countries which have enough resources from copper, since it shows a good economical advantage as serious competitor against copper.

The electrical conductivity of aluminum is high enough due to the huge number of free electrons rotating around its lattice structure [4]. However, the electrical conductivity of the commercially pure aluminum is higher than all aluminum materials and alloys. It has limited application because of its very low mechanical strength and toughness [5,6,7]. The demand for high strength and highly electrically conductive Al alloys for power transmission lines (e.g., wire and cable applications) has increased. Practically, by adding alloying elements to pure aluminum, aluminum’s strength can be significantly improved. However, on the other hand a great reduction in the electrical conductivity takes place due to the solute atoms and impurities generated by substitution of alloying elements. Another process affecting the electrical conductivity of aluminum is the heat treatment process, since elements in the solid solution phase represent a higher resistance than non-dissolved elements. That is why it is a great challenge to play with the strength of the pure aluminum in such a way that the decrease in its electrical conductivity will be still acceptable and valid for the selected application.

Since the electrical conductivity and the mechanical strength are most imperative properties for producing conductor materials, development of an aluminum conductor with a suitable combination from acceptable strength and high conductivity represents the main condition for using aluminum in electrical transmission cables. The electric conductivity property is affected by the metallic material’s microstructure, since it is very sensitive to the disturbance of electrons scattering due to any defects or solutes in the crystal structure. It has been seen for most age-hardened aluminum alloys that the relation between electrical conductivity versus hardness and tensile strength is “C-shaped” as reported by Hagemaier [8]. Initially, hardness of age-hardenable aluminum alloys decreases as the electrical conductivity increases because of the limited solubility in the solid solution phase, which affects the precipitation rate and generates many different phases. The opposite trend is valid at high temperature values, as low conductivity is associated with higher hardness values (which could be due to the dissolving back of the precipitates into the main matrix elements) [8].

Accordingly, recent studies and research activities have been trying to focus on developing high strength aluminum alloys with high electrical conductivity properties through novel processing and fabrication routes [9]. The improved properties can be achieved by many methods such as cold working, heat treatment, and adding alloying elements to the aluminum matrix. Addition of alloying elements including minor elements, major elements, and microstructure impurities can control the required strength and electrical conductivity of the alloy.

Alloying elements such as copper, magnesium and silver offer superb mechanical properties of the alloy at higher temperature values. Moreover, creep resistance is improved due to the uniform and fine precipitate distribution formed along the boundaries [10,11,12]. Additional benefits of copper additives include increasing the hardness and strength of aluminum casting alloys at all temperature levels and all heat treatment conditions, which leads to an improvement of the machinability of the fabricated alloys [13]. As a negative effect for adding copper as alloying elements, however, the low corrosion resistance of the aluminum-copper alloys increases the stress corrosion susceptibility in certain alloys and temperatures. Other alloying elements such as magnesium (Mg), have the potential to improve the hardness characteristics of aluminum alloys via substantial strengthening mechanisms. Thus, weldability, corrosion resistance and high mechanical strength can be achieved easily by using Mg additives.

Salihu et al. [14] studied the effect of adding Mg and ageing on the mechanical properties and microstructure of Al-Cu-Mg alloys. They reported that the increase in Mg percentage leads to an increase in hardness and tensile strength for the studied alloys; the addition of 2.5 wt% Mg can improve hardness by 23%, while tensile strength can be improved by 70%. They also investigated whether the addition of Mg has a good effect on ageing process due to grain refinement which reflect on the mechanical properties of the Aluminum alloy.

The effect of using silver into Aluminum alloys have been reported, but still quite little research articles are available in this field. The mechanical properties and microstructures of Al–Mg–Cu–Ag alloys are very sensitive to heat treatment parameters and deformation conditions [15,16,17]. The addition of silver to aluminum-4 wt%-copper alloy slows down the ageing rate of the low temperature limit, which provides a chance for hardening to take place by the expanding ageing time. More addition percentage of Ag to Al-Cu alloy will cause great precipitation hardenability [18] and high temperature stability [19,20]. Thus, adding Ag into Al-Mg-Cu alloy changes the phase formations conditions and slows down the degradation of the alloy at elevated temperatures levels [20]. The crystallographic orientation and chemical composition of Al-Cu-Mg alloys is affected by the addition of silver in the range of about 0.5% through artificial ageing, which improves the phase precipitation rate [20,21,22,23,24].

Earlier, Allen et al. worked on a few aluminum alloys and recommended a valuable relationship between tensile strength, hardness and electrical conductivity for 7079-T6, 7178-T6, 7075-T6, and 7002-T6 only. No such relation was found for other types of aluminum alloys [25,26,27]. Hagemaier reported that it was possible to find out the actual residual yield strength of 2024-T3/T4 and 7075-T6 alloys using hardness and electrical conductivity measurements [8].

To compare these overviewed alloys with other aluminum series alloys, it is observed that it has a very low strength, which we try to improve through this study. The major properties considered when choosing these alloys for structural application is their greater mechanical properties and electrical conductivity. In order to achieve this target, the current investigation will be focused on studying the electrical and mechanical properties of newly developed aluminum alloys, based on experimental design, varying chemical composition, cold working, heat treatment, and the aging process.

## 2. Experimental

The experimental work was carried out on pure Al and four different Al alloys (Table 1) produced by direct chill casting in our lab to select the best option out of them. The commercially available aluminum, copper, silver and magnesium were weighed, added to the pure Al powder, and then heated in the graphite crucible at 730 °C for 3 h to get the desired Al alloy. The chemical compositions of Al alloys (wt%) are given in Table 1. After heating, the molten metal is then poured into the pre-heated molds in order to get a solid as-cast sample 5 × 10 cm^2^ rectangle. The as-cast samples were then homogenized in a vacuum furnace at 540 °C for 24 h.

**Figure 1:**
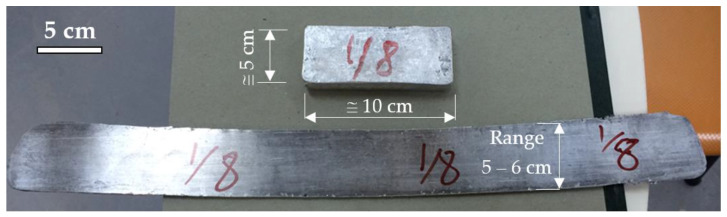
Alloys Before and after rolling.

After homogenization, the samples were hot rolled by heating the samples at 450 °C for 30 min maintaining the temperature of the rolls at 150 °C. The samples were hot rolled for multiple passes until 80–85% of the reduction ratio onto the sample was achieved. The samples were subjected to 7–8% of reduction in each pass and by interim heating of the sample in the furnace after each pass as shown in Figure 1.

### 2.1. Heat Treatment

**Figure 2:**
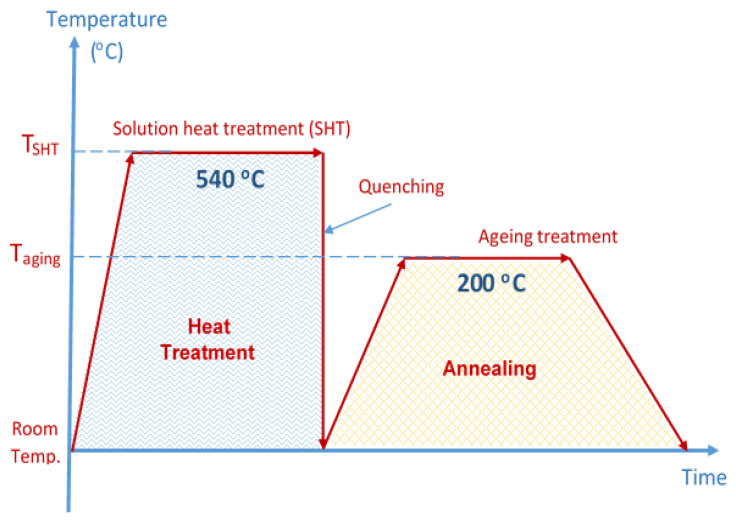
Schematic diagram for the heat treatment process applied.

Various heat treatments were used to study the behavior of the alloys and to assist the alloying elements which were segregated from aluminum during cooling down from melting phase while casting to get diffused and homogeneous solid diffusion of the alloying elements. The samples were heated at 540 °C for 30 min in vacuum furnace followed by quenching, air cooling and furnace cooling, or annealing as shown in Figure 2. The quenched samples were then aged at 200 °C for 1 h, 4 h, and 8 h, respectively.

The second step after heat treatment is annealing, in which the recrystallization effect can happen and to remove internal stresses. The obtained properties of the alloy are controlled by the its chemical composition, thickness of its cross section, and the cooling rate applied.

### 2.2. Mechanical Testing

Tensile specimens of the alloys were prepared by wire cutting in accordance with ASTM standards. The testing machine of INSTRON 5900 series was used with load cell capacity of 150 KN. The cross-head speed was fixed at 1.08 mm/min corresponding to 10–3 strain rate. The stress and strain were calculated using the load and displacement obtained from the machine. The tests were performed at room temperature. For hardness and conductivity measurements, square pieces (10 mm × 10 mm) of specimens with 2.5 mm thickness were cut.

### 2.3. Conductivity Measurement

From Ohm’s law, R = V/I, where V is the Voltage applied on the specimen, I is the passing current in Amp, resistance of the specimen towards electricity flow (R) can be calculated in Ohms (Ω). Inverse of resistivity is the electrical conductivity (σ) which is given as σ = 1/ρ. While resistivity (ρ in Ωm) can be obtained by using the equation: ρ = RA/L, where R is the resistance of the sample against to the electricity flow, A is the sample cross-sectional area in m^2^, and L is the sample length in meter.

The as rolled and heat-treated square pieces’ specimens were mirror polished prior to conductivity measurement. Conductivity was measured in a 4-point probe instrument (Model 6221, Keithley Instruments, Inc., Solon, OH, USA) which is a combined digital voltmeter and constant current source. The combination of four-points probe equipment is able to deliver a constant current source in order to measure volume resistivity or sheet resistance and resultant voltage. Resistivity of the alloys were measured and conductivity values were calculated.

## 3. Results and Discussions

**Figure 3:**
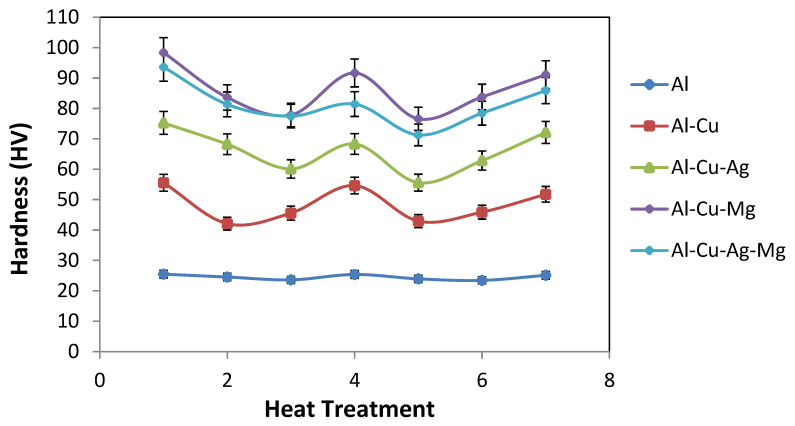
Variation of micro-hardness (HV) with heat treatment of different alloys.

Micro-hardness values of the as-rolled as well as the heat-treated aluminum alloys were given in Table 2 below. It can be seen that hardness values of as rolled product is higher than all heat-treated conditions for all alloys. The hardness of the as-cast sample is significantly affected by the heat treatment processes (Figure 3), with the tempered (aged) after 1 h sample having lowest hardness. The hardness also increased further after 8 h of aging treatment. With the exception of the quenched sample in which significant increase in hardness is attained, other heat treatment processes have little effect on the hardness characteristic of the alloys (Table 2).

**Figure 4:**
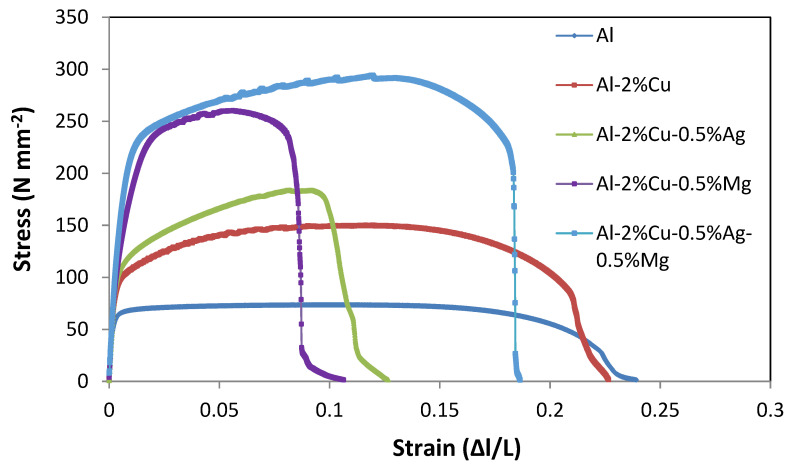
Stress–strain diagram of different alloys in as rolled conditions.

**Figure 5:**
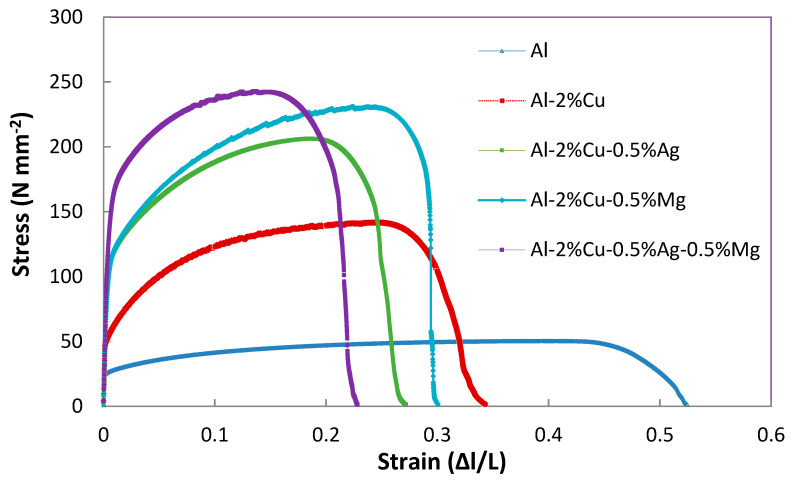
Stress–strain diagram of different alloys in age hardening conditions.

**Figure 6:**
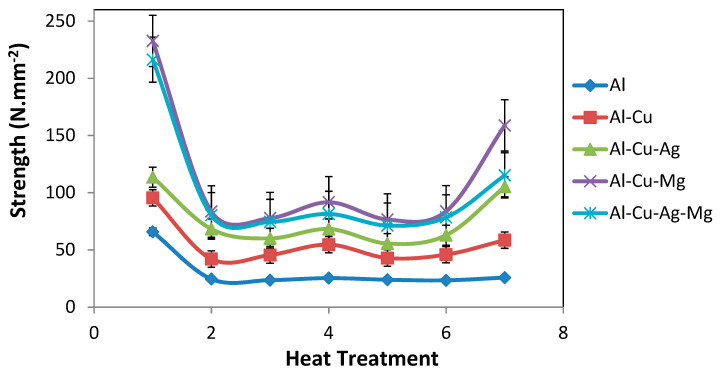
Variation of Strength (N/mm^2^) with heat treatment of different alloys.

Stress–strain diagrams were plotted as shown in Figure 4 and Figure 5 for as rolled and aging after 8 h condition and strength of the alloys were measured and presented in Table 3. The variations of strength of as rolled and different heat-treated conditions are shown in Figure 6.

It has been seen that the as-cast (as-rolled) specimens have the greatest yield strengths and ultimate tensile strength, then the age hardened specimens, quenched specimens, normalized and, in the last place, the annealed specimens. The presence of dislocations within the crystal structure due to the hot rolling process in the as-cast (as-rolled) structure allow it to get the highest strength and hardness among all of the samples, due to the brittleness effect gained by generating dislocations into the aluminum alloy. The variation in grain size after the heat treatment process for the remaining samples represents the main reason for low strength and hardness trend observed. That is exactly what Kenji et al. [28] concluded from their research work, which confirm that the grain refinement and the solid solution can contribute significantly to the aluminum–magnesium alloy hardening. Furthermore, it has been well reported in may previous researches that the large grained materials have less grain boundaries, and vice versa; fine grained materials have much more grain boundaries, and are thus stronger and harder than the large grained materials [29,30,31,32]. Another reason behind the higher hardness and toughness values for the age hardened samples over than quenched, normalized, and annealed samples is the dislocation motion during deformation, since it has more grain boundaries [33,34].

**Figure 7:**
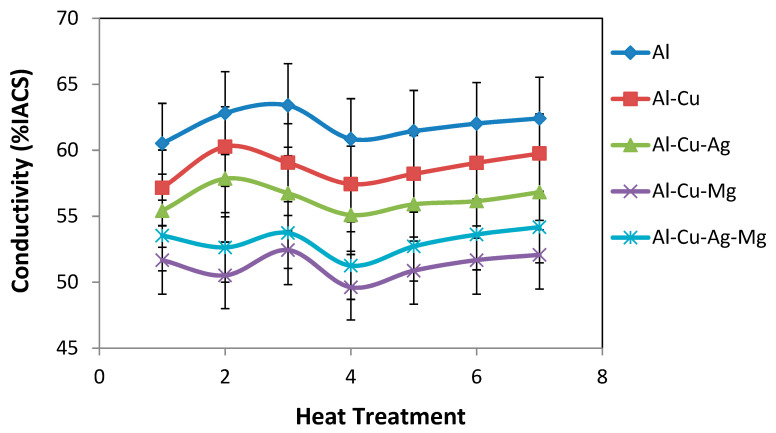
Variation of Conductivity (%IACS) with heat treatment of different alloys.

The electrical resistivity of all aluminum alloys in different heat-treated conditions was directly measured and the electrical conductivity was calculated and tabulated in Table 4. The conductivity values were compared for all heat treated as well as rolled samples as shown in Figure 7.

## 4. Conclusions

Four different grades of aluminum alloys were produced by varying composition of alloying elements viz. Cu, Ag & Mg. The as-cast alloys were then hot rolled followed by heat treatment in different conditions such as annealing, normalizing, quenching, and age hardening at different time zones. Strength and hardness of the as-cast (as-rolled) specimens are found to be higher followed by age hardened specimens with an exception of the quenched sample which exhibited substantial increase in hardness value. This condition in as-cast (as-rolled) specimens is caused by the existence of dislocations defects within the crystal structure of the aluminum alloys, which causes brittleness in the samples. On the other hand, the presence of larger grain boundaries or the grain growth after heat treatment are the reason for low strength and hardness in the heat-treated samples. In order to summaries the effect of alloying elements addition on the mechanical properties and electrical conductivity by specific values, it was found that by increasing the alloying elements content, yield strength results increased significantly by more than 250% and 500% for the as-rolled and 8 h-aged Al-Cu-Mg alloys, respectively. While the electrical conductivity reduces slightly with −14.6% and −16.57% for the as-rolled and 8 h-aged of the same Al-Cu-Mg alloy, respectively.

For the same alloy, the electrical conductivity of the age hardened sample was found to be greater than that of the other treatments. The increased electrical conductivity in the age hardened samples may be attributed to temperature changes and grain disassociation. As a result, the age hardened samples exhibited greater strength and conductivity. More research in this area is required to attain high electrical conductivity properties using innovative processing techniques and regulating the microstructural impurities in the alloy.

## Figures and Tables

**Table 1:** Pure Al and four different aluminum alloys composition.

Alloy	Al	Cu	Mg	Ag
1	100	-	-	-
2	Bal	2 wt.%	-	-
3	Bal	2 wt.%	-	0.5 wt.%
4	Bal	2 wt.%	0.5 wt.%	-
5	Bal	2 wt.%	0.5 wt.%	0.5 wt.%

**Table 2:** Hardness (HV) values of different heat-treated alloys.

Samples	As Rolled	Air Cooled Normalized	Furnace Cooled/Annealed	Quenched	Aging-1/Tempered	Aging-4	Aging-8
Al	25.47	24.53	23.60	25.37	23.93	23.43	25.10
Al-2%Cu	55.56	42.08	45.56	54.62	42.92	45.88	51.74
Al-2%Cu-0.5%Ag	75.23	68.23	60.10	68.30	55.57	62.83	72.10
Al-2%Cu-0.5%Mg	98.37	83.60	77.83	91.67	76.57	83.77	91.10
Al-2%Cu-0.5%Ag-0.5%Mg	93.60	81.33	77.47	81.43	71.27	78.43	85.87

**Table 3:** Yield Strength (N/mm^2^) values of different heat-treated alloys.

Samples	As Rolled	Air Cooled Normalized	Furnace Cooled/Annealed	Quenched	Aging-1/Tempered	Aging-4	Aging-8
Al	65.79	24.53	23.6	25.37	23.93	23.43	25.8
Al-2%Cu	95.48	42.08	45.56	54.62	42.92	45.88	58.54
Al-2%Cu-0.5%Ag	113.53	68.23	60.1	68.3	55.57	62.83	105.14
Al-2%Cu-0.5%Mg	232.64	83.6	77.83	91.67	76.57	83.77	158.78
Al-2%Cu-0.5%Ag-0.5%Mg	216.28	80.33	74.47	81.43	71.27	78.43	115.17

**Table 4:** Conductivity (%IACS) values of different heat-treated alloys.

Samples	As Rolled	Air Cooled Normalized	Furnace Cooled/Annealed	Quenched	Aging-1/Tempered	Aging-4	Aging-8
Al	60.53	62.82	63.4	60.87	61.46	62.03	62.42
Al-2%Cu	57.16	60.28	59.06	57.44	58.22	59.05	59.76
Al-2%Cu-0.5%Ag	55.42	57.85	56.75	55.1	55.91	56.15	56.82
Al-2%Cu-0.5%Mg	51.68	50.52	52.44	49.62	50.88	51.68	52.08
Al-2%Cu-0.5%Ag-0.5%Mg	53.54	52.64	53.74	51.26	52.72	53.62	54.18

## Data Availability

The data presented in this study are available on reasonable request from the corresponding author.
